# *ADRB2* and *ADCY9* Sequence Variations in Brazilian Asthmatic Patients

**DOI:** 10.3390/cimb46070414

**Published:** 2024-07-04

**Authors:** Viviane da C. Silva, Raquel L. de F. Teixeira, Rebecca E. E. N. O. do Livramento, Márcia Q. P. Lopes, Thyago Leal-Calvo, José E. Filho, Márcia B. V. Luduvice, Lilian de C. Rodrigues, Marcello Bossois, Patricia F. Schlinkert, Anderson S. Neves, Philip N. Suffys, José Roberto Lapa e Silva, Adalberto R. Santos

**Affiliations:** 1 Laboratory of Molecular Biology Applied to Mycobacteria, Oswaldo Cruz Foundation, Rio de Janeiro 21040-900, RJ, Brazil; vivicsbio@gmail.com (V.d.C.S.); adalbertorezende@yahoo.com.br (A.R.S.); 2 Leprosy Laboratory, Oswaldo Cruz Foundation, Rio de Janeiro 21040-900, RJ, Brazil; 3 Departamento de Clínica Médica, Faculdade de Medicina, Federal University of Rio de Janeiro, Rio de Janeiro 21941-617, RJ, Brazil; 4 Pneumology Department, Clementino Fraga Filho University Hospital, Federal University of Rio de Janeiro, Rio de Janeiro 21941-617, RJ, Brazil; 5 Projeto Brasil sem Alergia, Duque de Caxias 25070-350, RJ, Brazil; 6 Institute of Thoracic Medicine, Clementino Fraga Filho University Hospital, Federal University of Rio de Janeiro, Rio de Janeiro 21941-617, RJ, Brazil; jrlapa@hotmail.com

**Keywords:** *ADRB2*, *ADCY9*, asthma, pharmacogenetics, polymorphisms

## Abstract

Asthma is a chronic inflammatory respiratory condition, characterized by variable airflow limitation, leading to clinical symptoms such as dyspnea and chest tightness. These symptoms result from an underlying inflammatory process. The β2 agonists are bronchodilators prescribed for the relief of the disease. Nevertheless, their efficacy exhibits substantial interindividual variability. Currently, there is widespread recognition of the association between specific genetic variants, predominantly located within the *ADRB2* and *ADCY9* genes and their efficacy. This association, usually represented by the presence of non-synonymous single nucleotide polymorphisms (SNPs) have a strong impact in the protein functionality. The prevalence of these mutations varies based on the ethnic composition of the population and thus understanding the profiles of variability in different populations would contribute significantly to standardizing the use of these medications. In this study, we conducted a sequence-based genotyping of the relevant SNPs within the *ADRB2* and *ADCY9* genes in patients undergoing treatment with bronchodilators and/or corticosteroids at two healthcare facilities in the state of Rio de Janeiro, Brazil. We investigated the presence of c.46A>G, c.79C>G, c.252G>A, and c.491C>T SNPs within the *ADRB2*, and c.1320018 A>G within the *ADCY9*. Our results were in line with existing literature data with both for individuals in Brazil and Latin American.

## 1. Introduction

Asthma is a chronic inflammatory disease of the respiratory tract that affects approximately 300 million people of all ages, genders, and ethnic groups worldwide [1,2]. The main characteristic of the disease is airflow limitation due to increased mucus production, and the thickening and narrowing of the airways [3]. Its treatment is based on three main classes of drugs, namely corticoids, β2-agonists, and leukotriene inhibitors. The main objective of treatment is to minimize airway obstruction and reduce the patient’s risk of death [4]. However, treatment success is typically less than ideal in many patients. Even in patients with apparently identical clinical phenotypes, the response to treatment may vary [5]. Several factors can influence the way the patient will respond to treatment, including the misuse of prescribed medications by patients, the duration of treatment, poor communication between doctor and patient, and the limitations of the healthcare system [6]. According to Ortega and Bleecker (2012), genetics can contribute up to 70% of this interindividual variability, which corresponds to the maximum limit of genetic variation [7]. This observation indicates that a clinically relevant part of the response to the major classes of asthma medications may be due to genetic differences.

Introduced as asthma treatment in the 1950s, the response to β2-agonists varies considerably according to the studied group [8]. Some authors suggest that this variation may be related to polymorphisms in two genes involved in drug pharmacodynamics, *ADRB2* and *ADCY9* [9].

The β2-adrenergic receptor (ADRB2) is a transmembrane G protein-coupled receptor composed of 413 amino acid residues [10]. It is found in the airways, mast cells, epithelial, endothelial, and type II cells [11], and β2-agonist drugs selectively interact with this receptor. Once the receptor binds to the drug, its β subunit dissociates from the G protein and triggers a signaling cascade [12]. Downstream of the ADRB2 receptor is the protein adenyl cyclase 9 (ADCY9), an integral membrane protein of 1353 amino acid residues. This protein is activated by the previously dissociated G protein from ADRB2. Once active ADCY9 catalyzes the conversion of adenosine triphosphate (ATP) to cyclic adenosine monophosphate (cAMP). The cAMP activates protein kinase A, which in turn stimulates Ca2+/Na+ exchange, resulting in a reduction in intracellular calcium levels, ultimately leading to bronchodilation [13]. 

The ADRB2 receptor is encoded by the *ADRB2* gene, an intronless gene of approximately 2033 bp located on the long arm of chromosome 5 (5q31-33) [14]. It is a highly polymorphic gene, but most studies focus mainly on the following three non-synonymous Single Nucleotide Polymorphisms (SNPs): c.46A>G which results in a modification in the amino acid sequence, specifically in codon 16, exchanging an arginine for a glycine, (Arg16Gly); c.79C>G which generates the exchange of a glutamine for a glutamate at codon 27 (Gln27Glu); and c.491C>T, which is less frequent and results in the exchange of the amino acid threonine for an isoleucine in codon 164 (Thr164Ile). There are many controversies regarding the role of these mutations in the response to treatment. For position c.46A>G, while some authors have found that there is a greater response in AA homozygotes, others have found that the response is better in patients who are GG homozygotes [15,16,17]. Some authors support the hypothesis that mutations in the c.79C>G position do not interfere with the response, while others have shown that CC genotype carriers present a better response when compared to the others [18,19]. Regarding the c.491C>T, this SNP occurs close to the β2 agonist binding site, which causes a decrease in the interaction between the ligand and the receptor and between the receptor and the ADCY9 protein. Studies have already demonstrated that the mutation is associated with a lower response to treatment with β2 agonists [20]. 

These outcomes, however, were also analyzed from another aspect. Functional analysis performed based on the formed haplotypes rather than the genotypes of individual SNPs is also very informative. It was observed, for example, that these two mutations are in a strong linkage disequilibrium in which the Gly16/Gln27 haplotype was associated with bronchial hyperresponsiveness [21] and the ArgGly/GluGlu haplotype showed a decrease in the negative regulation promoted by the β2 agonist in the airway smooth muscle cells [22]. The Thr164Ile polymorphism is located in the fourth transmembrane domain of the receptor. This domain is where the pocket for the binding of agonists is formed [20] and, in an in vitro study, receptors with the mutant variant showed an affinity for the agonist four times lower than receptors with the wild-type variant [23]. 

ADCY9 is encoded by the *ADCY9* gene which is located on the short arm of chromosome 16 (16p13.3). For this gene, the most studied polymorphism is c.1320018A>G (rs2230739). Small and collaborators demonstrated that this SNP causes a loss of protein function when compared to the wild type, as well as a decrease in activity after G protein stimulation [24]. Studies carried out in different populations obtained similar results by demonstrating that the polymorphism increases the bronchodilator response in asthmatic patients who use β2 agonists associated with inhaled corticosteroids [25]. 

The scientific community is aware that the frequency of these SNPs may vary according to the ethnic group studied. According to the 1000 genomes database [26], for the mutation c.46A>G, the frequencies of the minor allele are 0.47, 0.61, and 0.54 for African, European, and South Asian populations, respectively; however, the literature data also show frequencies of 0.39 for American Caucasians and 0.49 for American Blacks [27,28]. For the c.79C>G mutation, these frequencies are 0.13, 0.41, and 0.19 for the same populations, respectively. The c.491C>T mutation is rare in several populations and in this database, it does not appear in African populations. For Europeans, the frequency of the minor allele is 0.018 and in South Asia, it is 0.033. With regard to the c.1320018A>G mutation for the *ADCY9* gene, the frequencies are 0.11 in the African population, 0.34 in the European population, and 0.24 in South Asia.

It is known that the Brazilian population is composed of the miscegenation of three parental groups (African, Amerindian, and European); however, although we also experience a large variance in response to β2-agonists, little is known about the frequency of these mutations. Therefore, the objective of this study was to investigate the distribution of the c.46A>G, c.79C>G, and c.491C>T (rs1042713; rs1042714 and rs1800888) *ADRB2* and c.1320018A>G (rs2230739) *ADCY9* polymorphisms in asthmatic patients from two different healthcare facilities in the state of Rio de Janeiro, Brazil.

## 2. Materials and Methods

### 2.1. Patients

After obtaining written consent, 155 unrelated patients diagnosed with asthma based on the clinical criteria were enrolled in this study. Among these participants, 31 were recruited from the ‘Projeto Brasil sem Alergia’ (PBSA/Alergorio), while the remaining 124 individuals were selected from the asthma outpatient clinic at the Institute of Thorax Disease (IDT), Federal University of Rio de Janeiro.

The inclusion criteria for this study involved individuals aged 18 and above who, regardless of gender, had been diagnosed with asthma and were currently receiving treatment with bronchodilators and/or corticosteroids. The study received ethical approval from the Ethics and Research Committee of the Oswaldo Cruz Foundation (CEP-Fiocruz) on 14 November 2018, under reference number 3.021.497.

### 2.2. Sample Collection and DNA Extraction

A volume of 5 mL of peripheral blood was collected from each patient in a “Vacutainer” tube containing citrate buffer or EDTA as an anticoagulant agent. DNA extraction was performed from 200 µL of peripheral blood using the commercial kit “QIAamp DNA blood^®^” (Qiagen Inc., Germantown, MD, USA), following the manufacturer’s instructions. Quantitative and qualitative analysis of the extracted DNA were determined by means of measurements in a fluorometer, Qubit 3.0 (Invitrogen™ Qubit™ 3 Fluorometer, Carlsbad, CA, USA).

### 2.3. Genotyping

Genotyping for both genes was achieved by polymerase chain reaction (PCR) and automated sequencing based on the Sanger method [29]. For ADRB2, a 710 pb fragment, of the coding region, was amplified by PCR, using primers 5′CACCACAGCCGCTGAATGAGG3′ (forward) and 5′GGCTTTGGTTCGTGAAGAAGTC3′ (reverse). For the ADCY9 gene, the primers 5′GGGGTAGTAGAGGGAGACAGC3′ (forward) and 5′AGCTGAAGGTGGGACTAGCA3′ (reverse) were used to amplify a 206 pb fragment in exon 7. PCR reactions were performed in 50 µL of reaction mix containing 50 ng of genomic DNA, 1.5 mM MgCl_2_, 0.4 mM dNTPs, 1X PCR buffer, 1 U of recombinant Taq DNA polymerase (Invitrogen™, Carlsbad, CA, USA), and 0.2 µM of each primer for *ADRB2* or 0.4 µM of each primer for *ADCY9*. Samples were incubated at 94 °C for 5 min, followed by 35 cycles of 94 °C for 1 min, 62 °C for *ADRB2* or 67 °C for *ADCY9* for 1 min, and then 72 °C for 90 s. The final extension took place at 72 °C for 5 min. 

### 2.4. Sequence Analysis and Statistics

We used the SeqScape v.2.5 software (Applied BioSystems, Foster City, CA, USA) to analyze each sequence and identify the SNPs. Sequence data of each sample were aligned with the reference sequences NG_016421.1 for *ADRB2* and NG_011434.1 for *ADCY9*. The genotype and allele frequencies of polymorphisms were determined, and deviations from the Hardy–Weinberg equilibrium were calculated by the Hardy–Weinberg exact balance test [30] with SNPassoc v.2.0-11. Haplotype reconstruction for the *ADRB2* gene was performed using the PHASE v2.1.1 software through a Bayesian approach in order to obtain unambiguous genotypes and determine the most likely pair of alleles.

All the PCR products were purified using the commercial PureLink^®^ PCR Purification Kit (Invitrogen™, Carlsbad, CA, USA), according to the manufacturer’s instructions. The sequencing reaction was performed using the aforementioned primers and the ABI PRISM Big Dye Terminator Kit v. 3.1 Kit (PE Applied BioSystems, Foster City, CA, USA) according to the manufacturer’s recommendations on an ABI PRISM 3730 DNA Analyzer (PE Applied BioSystems, Foster City, CA, USA).

## 3. Results

### 3.1. Characteristics of the Population

Samples were obtained from a total of 155 asthma patients, representing various asthma phenotypes. The age ranged from 18 to 90 years, with a mean age of 56.14 ± 16.19 years. The study included both genders, all of whom were residents of Rio de Janeiro. Among these patients, 124 (80%) were recruited from IDT, while the remaining 31 (20%) were from PBSA (Table 1). A higher number of female patients were randomly enrolled compared to males. The phenotype of severe asthma was predominant among the study population.

### 3.2. ADRB2 Genotyping: SNP Identification, Allele and Genotype Frequencies

Among the 155 patients studied, sequencing of PCR-amplified DNA samples revealed four SNPs (c.46A>G, c.79C>G, c.252G>A, and c.491C>T) within the *ADRB2* gene. Of these, three were non-synonymous SNPs, and one was a synonymous SNP, all of which had been previously described. The genotype and allele frequencies of these SNPs are described in Table 2. Notably, the c.46A>G SNP was found to be the most frequent, while the c.491C>T SNP was the rarest.

Using the PHASE v2.1.1 software, we inferred six distinct haplotypes, and their frequencies are described in Table 3. Among the population studied, haplotypes 4 and 6 were found to be the most frequent.

### 3.3. ADCY9 Genotyping: SNP Identification, Minor Allele and Genotype Frequencies

Sequence analysis of 153 DNA samples for the *ADCY9* gene revealed the presence of a previously described polymorphism, c.1320018A>G. The genotypic and allele frequencies of this polymorphism are presented in Table 4. Interestingly, it is noteworthy that the mutant allele appears to be more prevalent in heterozygous individuals compared to homozygous individuals at this position.

## 4. Discussion

In this study, we evaluated the profile of the known polymorphisms within the *ADRB2* and *ADCY9* genes of pharmacogenetic interest for the treatment of asthma. The study population consisted of asthma patients from two treatment units in the state of Rio de Janeiro who were under treatment with bronchodilators and/or corticosteroids.

The patients were randomly selected for a determined period of time, after which the female patients represented the majority. This finding is consistent with that of Menezes and Marques [31,32], who revealed that the prevalence and incidence of asthma are higher in women than in men in adulthood by several factors, such as hormonal influence, exposure patterns, healthcare seeking behavior, among others.

It is well established that the prevalence of *ADRB2* polymorphisms varies based on the specific ethnic group under investigation [28]. Given the rich ethnic diversity of the Brazilian population and the importance of standardizing asthma treatment, our study focused on evaluating the prevalence of the three primary pharmacologically relevant polymorphisms in this gene. However, after sequencing, we found an additional synonymous polymorphism, c.252G>A (rs1042717), with a relatively high frequency within the coding region. This SNP has already been described in Latino and North American cohorts [33,34]. Given the limited availability of information on SNP profiles in the Brazilian population, together with the high prevalence of asthma, considerable variability in response to reliever medications (bronchodilators and corticosteroids), and the complex ethnic diversity, particularly in the southeastern region of Brazil, we recognize the importance of providing comprehensive variability profiles for these genes. Through sequencing, we genotyped samples obtained from patients living in this region and made all genotype, allele, and haplotype frequency data accessible.

We observed that the most common polymorphism was c.46A>G, whereas c.491C>T was the least frequent. The elevated frequency of the c.46A>G SNP in our study aligns with the findings of Mattevi et al., who studied a population from the southern region of Brazil where European ancestry predominates. However, in terms of genotype distribution, while our sample displayed a homogeneous distribution, the southern region’s population showed an increased frequency of the heterozygous genotype [35]. In a study of an African-American cohort, Xie and colleagues reported a frequency of 52.2% for the mutant allele at this position, which closely aligns with the frequencies observed in our study [36].

In relation to the c.79C>G mutation, our data align with those observed in populations that have identified this mutation as the second most frequent in the *ADRB2* gene. Furthermore, these populations have reported high frequencies for the mutant allele. Specifically, we identified a frequency of 0.2 for the mutant allele at this position, which closely resembles the findings reported by Maxwell in a cohort of Saudi individuals and by Xie in African-Americans [28,36].

Certainly! The *ADRB2* gene has been found to be associated with various diseases beyond asthma, including obesity, chronic obstructive pulmonary disease, and certain types of cancer [37,38,39]. In this study, we observed high frequencies of the mutant allele at positions c.46A>G and c.79C>G. These findings may not be specifically related to asthma treatment but could be linked to some adaptive advantage that has not been investigated here, possibly associated with favorable outcomes in these other diseases. Similarly, the high frequency of heterozygotes suggests that the presence of both alleles may result in a phenotype that is more advantageous than those generated by the expression of either allele in isolation.

The c.491C>T polymorphism, which was also observed in our study, is notably rare in many populations. In our study, the frequency of homozygous mutants for this polymorphism was 0.06. The minor allele frequency of 0.02 aligns with the frequency found in the Sudanese population [28].

Previous studies have indicated that synonymous SNPs in the *ADRB2* gene can potentially affect RNA stability and thus impact the protein levels [40]. The mutation at position c.252G>A, being a synonymous SNP, has received limited research. However, in our population, it occurred at a frequency of 0.2 and contributed to the formation of the most frequent haplotype.

Considering this, it is essential to verify the presence of this allele in other populations and conduct association studies that include this particular SNP. Our frequency data align with the findings of Martínez-Aguilar and colleagues in a cohort of Latino individuals residing in the United States [34]. The similarity in frequencies between our population and the Latin population can be attributed to their shared ancestral composition, specifically the presence of Amerindian ancestry.

In general, the frequencies found in our study for the SNPs of the *ADRB2* gene are very close to those found for the same SNPs in cohorts from the African continent, which reaffirms the ancestral parental composition of our population, indicating that the occurrence of these mutations is probably anterior to the migratory processes that happened during the colonization process of Brazil. 

In our study, the frequencies of the *ADRB2* gene SNPs closely resemble those found in cohorts from the African continent. This reaffirms the ancestral parental composition of our population, suggesting that these mutations likely predate the migratory processes that occurred during the colonization of Brazil.

Comparing the frequency of the *ADCY9* c.1320018A>G SNP in our study with three distinct populations (Caucasians, Asians, and African Americans) previously studied, we observed consistency, particularly in the high frequency of the heterozygous genotype [24], which is also reflected in our population. The frequency of the mutant allele varies between 0.16 in the African-American population and 0.37 in the Asian population.

In another study conducted in a population from Bahia, located in the northeast of Brazil where individuals of African descent predominate, a similar allele frequency lower than 0.2 was observed, consistent with our findings [41]. Furthermore, the high frequency of heterozygous genotypes contributes to the maintenance of genetic diversity in this population and may consequently lead to a better response to selective necessities.

In our population, the most frequent haplotypes identified were haplotype four (GCAC) with a frequency of 0.26, and haplotype six (GGGC) with a frequency of 0.21. At the time of our study, there were no references to haplotype four in the literature, while haplotype six had been extensively studied. It is worth noting that the two mutations present in haplotype six are in linkage disequilibrium.

A study conducted with Italians reported a frequency of 0.31 for the Gly16–Glu27 genotype, which is part of haplotype six [21]. The high frequency of these haplotypes may suggest potential changes in the expression and/or activation of the ADRB2 gene. However, further studies are needed to evaluate this hypothesis and gain a better understanding of the functional implications.

## 5. Conclusions

It is important to note that the frequencies of polymorphisms in the *ADRB2* and *ADCY9* genes can vary within the same territory based on the ethnicity under study.The Brazil is a country of continental dimensions and significant ethnic diversity, the population exhibits regional variations in terms of miscegenation, with areas such as the southern region showing less pronounced mixing.

The genetic variants examined in this study have pharmacological implications as they have been associated with varied responses to medication used in the management of various diseases, including asthma. In our study, we observed relatively high frequencies of mutant alleles for the investigated SNPs, suggesting a potential adaptive advantage conferred by these alleles. The prevalence of these alleles in the Brazilian population remains relatively unexplored, emphasizing the need to further investigate the distribution of these mutations in other cohorts of Brazilians. This not only serves to describe the polymorphisms present in the Brazilian population but also evaluate their impact on the treatment of asthma and other diseases.

## Figures and Tables

**Table 1:** Clinical and demographic characterization of the study population.

	IDT *N* = 124	PBSA *N* = 31	Total
Gender			
Female	102	24	126
Male	22	7	29
Mean Age	58	48.5	56.1
Asthma Phenotype			
Light	28	6	34
Moderate	41	14	55
Severe	55	11	66

**Table 2:** Genotype and minor allele frequencies of the *ADRB2* SNPs.

Position	*N* = 155	Genotype Frequency (%)	MAF *
c.46A>G			
AA	51	32.9	0.5
AG	52	33.54	
GG	52	33.4	
c.79C>G			
CC	107	69.03	0.2
CG	30	19.35	
GG	18	11.61	
c.252G>A			
GG	85	54.83	0.2
GA	51	32.90	
AA	19	12.25	
c.491C>T			
CC	150	96.77	0.02
CT	4	2.58	
TT	1	0.64	

* MAF = Minor allele frequency.

**Table 3:** Identification of haplotypes and their respective frequencies.

Haplotype	*N* = 310 *	c.46A>G	c.79C>G	c.252G>A	c.491C>T	Frequency (%)
1	152	A Arg	C Gln	G	C Thre	49.03
2	2	A Arg	C Gln	A	C Thre	0.64
3	3	G Gly	C Gln	G	C Thre	0.96
4	81	G Gly	C Gln	A	C Thre	26.12
5	6	G Gly	C Gln	A	T Ile	1.93
6	66	G Gly	G Glu	G	C Thre	21.29

* Number of alleles analyzed.

**Table 4:** Genotype and allele frequencies for the *ADCY9* gene.

Genotype	*N* = 153	Genotype Frequency (%)	MAF *
c.1320018A>G			
AA	92	60.13	0.24
AG	48	31.37	
GG	13	8.49	

* MAF = Minor allele frequency.

## Data Availability

All data generated or analyzed in this study are available from the. corresponding author on reasonable request.
